# Coupled Benchtop NMR and EPR Spectroscopy Reveals the Electronic Structure of Viologen Radicals in a Redox Flow Battery

**DOI:** 10.1021/acselectrochem.5c00194

**Published:** 2025-09-23

**Authors:** Giu A. Silva Testa, Mathijs A. Damhuis, Tom Speelman, Kim Baas, Johannes A. A. W. Elemans, Evan Wenbo Zhao

**Affiliations:** † Magnetic Resonance Research Center, Institute for Molecules and Materials, 614702 Radboud University, 6525 AJ Nijmegen, The Netherlands; ‡ Theoretical and Computational Chemistry, Institute for Molecules and Materials, Radboud University, 6525 AJ Nijmegen, The Netherlands; § Spectroscopy and Catalysis, Institute for Molecules and Materials, Radboud University, 6525 AJ Nijmegen, The Netherlands

**Keywords:** *operando* NMR, *operando* EPR, redox flow battery, viologen

## Abstract

Viologens are a class of organic molecules with promising properties for redox flow battery applications. However, their molecular-level mechanisms remain challenging to fully probe and understand. In particular, the role of π-dimerization of singly reduced radicals is still debated, with conflicting views on its impact on battery performance. The electronic structure of a viologen radical in a working redox flow battery has remained elusive. Magnetic resonance spectroscopies offer powerful methods for studying flow batteries in *operando*, but their high cost and maintenance requirements make them less accessible to many researchers in the community. In this study, we introduce a novel dual benchtop nuclear magnetic resonance and electron paramagnetic resonance methodology to investigate viologen-based redox flow batteries. We revealed the electron spin density of in situ generated radicals via the hyperfine coupling interactions measured by EPR, aided by DFT calculations. Notably, the relatively low radical concentration observed during battery cycling suggests that π-dimers form even at a low concentration of 10 mM. Stability of the viologen radicals was also monitored. This study highlights the strength of the dual benchtop approach in uncovering molecular-scale processes in a redox flow battery. Moreover, its flexible and accessible design makes this coupled benchtop technique a versatile tool for investigating a wide range of flow electrochemical systems.

## Graphical abstract

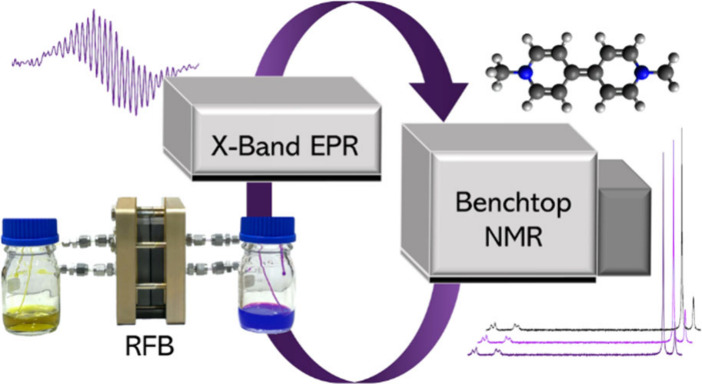

## Introduction

As global energy demand continues to rise, there is a pressing need to adopt renewable energy sources, such as wind and solar power.^1^ Nevertheless, the intermittent nature inherent to renewable sources of energy demands the advancement of reliable, sustainable and low-cost energy storage technologies to bridge the gap between generation and consumption. Electrochemical energy storage, in particular, boasts a myriad of advantages, from its environmentally friendly operation to its flexible power and energy profiles.^2^ Among these technologies, redox flow batteries (RFBs) have emerged as a promising solution due to their modular design and flexible scaling capabilities.^3^ As of now, vanadium-based RFBs stand out as the most commercially available example, owing to their stability and extended cycle life.^4^ However, the limited availability and supply risks associated with vanadium have driven research efforts toward alternative redox-active materials,^5^ particularly organic-based electrolytes. Despite promising developments, new chemistries must demonstrate enhanced energy densities and prolonged lifespans to be viable alternatives.^6^ These performance metrics are linked to the stability and concentration of redox-active species in the electrolyte, making a deep mechanistic understanding of their electrochemical behavior essential. Intrinsically, the energy density of a RFB is associated with the number of electrons stored per molecular component and the concentration of these molecules in the electrolyte. Battery lifespan is directly tied to the longevity of its components, with the stability of redox-active molecules playing a critical role.^7^ To design a battery with an optimized overall performance, the reaction mechanisms by which the redox-active molecules are interconverting between their various redox states needs to be fully comprehended. Achieving a molecular-level understanding, particularly under realistic battery operating conditions, involves thoughtfully devising non-invasive characterization methods.

In this regard, Nuclear Magnetic Resonance (NMR) and Electron Paramagnetic Resonance (EPR) spectroscopies can be formidable tools for characterizing different phenomena.^8^ Both techniques can provide molecular-level insights into the composition and dynamics of the electrolyte molecules, and thus insights into the reaction mechanisms.^6,9^ Bearing in mind the inherent flow nature of RFBs and considering the non-invasive and quantitative capabilities of these techniques, both NMR and EPR can be harnessed for inline *operando* detection, that is, the liquid negolyte or posolyte solutions can be flowed into the instruments and characterized while the battery is operating, thus obtaining real-time data of the chemical reactions.

While *operando* high-field NMR has been increasingly applied to study RFBs,^10–12^ its widespread adoption is hindered by the high cost, space requirements, and maintenance demands of superconducting magnets. A more accessible and practical alternative is the use of benchtop NMR systems, which, when coupled with EPR, can provide complementary structural and electronic insights in a compact and cost-effective setup. Prior work demonstrated the feasibility of combining high-field NMR with benchtop EPR for monitoring anthraquinone-based RFBs.^6,13^ Remarkably, even benchtop NMR has proven effective for probing processes in RFBs, such as degradation, crossover, and molecular aggregation.^9,14^ However, the potential of a fully benchtop dual NMR-EPR setup for *operando* characterization remains, until now, unexplored. In this study, we report for the first time a dual *operando* benchtop EPR and NMR set-up for monitoring a RFB based on viologen chemistry. By simultaneously tracking the evolution of ^1^H-NMR signals and EPR-active radical species, we aim to provide direct spectroscopic evidence of the battery processes and radical formation in an aqueous RFB.

Viologens, or doubly alkylated 4,4′-bipyridine derivatives, are a class of redox-active organic compounds known for their relatively high solubility in neutral aqueous media, synthetic tunability, good redox cyclability and stability, and fast reaction kinetics.^15–17^ These compounds can undergo one or two-electron redox processes per molecule, which makes them one of the promising negolyte candidates in RFBs.^16^ Although viologens generally possess desirable characteristics, their electrochemical performance can be limited by a combination of factors. Notably, the first reduction step of viologens generates highly reactive radical cations, which are ready to transfer electrons to molecular oxygen, forcing the battery to operate under inert atmospheres.^18,19^ Moreover, these radicals are prone to self-assembling via π-dimer formation, which has been previously reported to negatively impact the battery’s capacity.^20–22^ Fortunately, the design of bipyridinium structures can be tailored to counteract these challenges. This can be achieved by different measures, for example, by introducing steric hindrance or modifications to the overall skeletal structure of the system, which can result in the inhibition of the undesired π-stacking interactions.^23–26^ However, a recent study on associating bipyridinium molecules suggested that π-dimerization can actually *suppress* the reactivity of oxygen-sensitive radicals, leading to higher capacity retention and improved air tolerance.^12^ The apparent discrepancy in the role of dimerization highlights the need for further investigation of viologen chemistry, to better understand the impact of radicals on battery performance. Despite the extensive exploitation of the viologen family for RFBs, the electronic structure of a viologen radical has remained elusive, due to the challenge of resolving the hyperfine coupling between the unpaired electron and neighboring nuclei at high concentrations. Here we set out to apply our new benchtop methodology to reveal the electronic structure of a viologen molecule in a working RFB.

## Experimental Section

Amongst the different existing viologens, methyl viologen dichloride (MV^2+^) was chosen as the subject of this study due to its structural and synthetic simplicity.^15,16,27–29^ The synthetic procedure and NMR spectra of MV^2+^ are included in the Supporting Information (SI) (Figures S1–S3). We chose 4-hydroxy-2,2,6,6-tetramethylpiperidin-1-oxyl (TEMPOL) as the posolyte counterpart. The corresponding redox reactions for both electrolytes are depicted in Figure 1. It is worth noting that only the first reduction of MV^2+^ to MV^+•^ is accessed under aqueous conditions in our study, as the fully reduced form MV^0^ has neutral character and a tendency to precipitate.^27^ Both electrolytes were separated by an anion exchange membrane.

**Figure 1:**
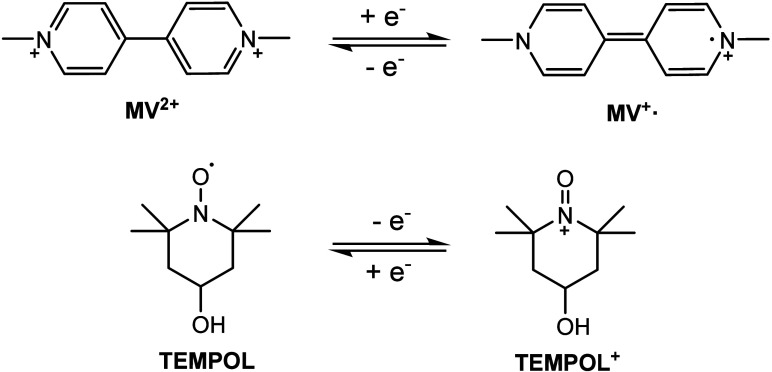
Schematic representation of the redox reactions, structures, and nomenclature of the compounds used within this study. MV^2+^ has two Cl^–^ counterions (not shown). The cyclic voltammograms for both electrolytes are presented in Figure S4.

Our coupled NMR and EPR setup including the devices necessary for RFB operation is shown in Fig. 2a. The RFB cell was placed outside the two magnets. The cell’s assembly is described in the SI following a similarly reported procedure.^9^ The EPR (X-Band) and NMR (80 MHz) instruments were positioned side by side and connected with 1/16 in O.D. tubing to their respective flow sampling tubes, which allows the solution of interest to circulate through the entire system. For this purpose, we modified the EPR tube, as described in detail in the section *Flow System* in the SI (Figure S5). The NMR flow apparatus was used as provided by the manufacturer. A proper illustration with the flow direction through the instrumentation is shown in Figure S6. Specifications of all tubing and connectors are tabulated in Table S1, and a comparison between benchtop and high-field NMR systems is given in Table S2. This configuration allows for the real-time spectroscopic analysis of the electrolyte of choice. For the experiment described in this paper, the setup requires an accessible nitrogen gas line to operate in inert atmosphere, since MV^+•^ formed upon the electrochemical reduction is sensitive to air.^30^ An inert atmosphere was achieved by bubbling nitrogen into both sealed reservoirs for 30 min prior to battery operation.

**Figure 2:**
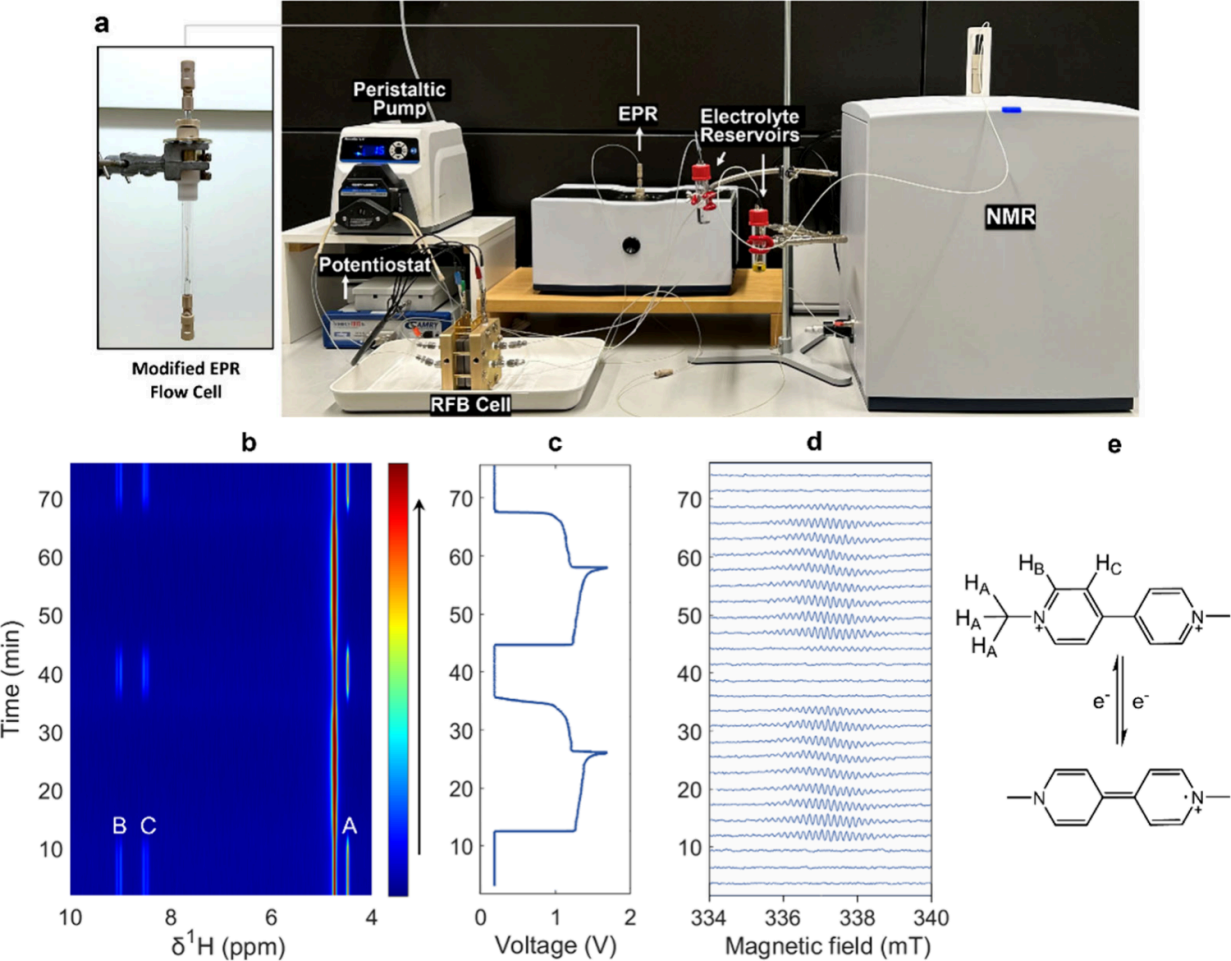
(a) Dual coupled benchtop NMR and EPR set-up for electrochemical characterization. A peristaltic pump allows for the flow of the electrolyte solutions to be circulated through the RFB cell and the rest of the system. The EPR sampling tube is depicted outside of its cavity to display the manually attached connectors, which allow for the attachment of the tubing. (b) Inline *operando* pseudo-2D ^1^H-NMR spectra, (c) voltage profile, and (d) continuous wave EPR spectra during battery cycling of a full RFB cell containing 10 mM MV^2+^ against 20 mM TEMPOL in D_2_O. The color scaling in (b) indicates the relative intensity of the proton resonances in arbitrary units, red: strong; blue: weak. The protons of MV^2+^ are labelled in the molecular scheme in (e). The HDO signal originates from the proton exchange between the residual water in commercially obtained deuterium oxide (98%).

## Results and Discussion

*Operando* experiments were performed using deuterated aqueous solutions of MV^2+^ (10 mM, in 20 mL) and TEMPOL (20 mM, in 20 mL), with sodium chloride as a supporting electrolyte (0.1 M). The electrochemical procedure consisted of two charge–discharge cycles. Figure 2b-d presents the time-synchronized electrochemical data alongside the NMR and EPR spectra. A first glance reveals a strong correlation among all three measurements. Initially, the battery was set to rest at a voltage of 0.2 V, at which the two aromatic doublets (H_C_ and H_B_) and methyl singlet (H_A_) of MV^2+^ are visible in the NMR spectra. During charging at a constant current of 20 mA (current density of 4 mA·cm^–2^), the battery voltage increased gradually up to a cut-off value of 1.7 V, at which MV^2+^ underwent a one-step reduction. Throughout this period, the proton resonances in the NMR spectra rapidly faded, accompanied by a broadening of the HDO signal (= 4.74 ppm). Both events can be explained by the emergence of radical cations, which can be directly observed by the appearance of a new signal in the EPR spectra.

The unpaired electron spins of the radicals cause strong local magnetic field fluctuations that shorten the nuclear spin relaxation times, leading to severe broadening and eventual loss of NMR signals of the radical cations. It is worth noting that NMR only can detect the diamagnetic (non-radical) species present. The rapid loss of proton signals of all species in the solution upon onset of charge was caused by the intermolecular electron transfer between the radical species and its oxidized form.^6,9^ This phenomenon is reversed by the end of the discharge period, where the EPR signal intensity decreased over time as the radical was consumed. Finally, the initial proton signals reappeared in the ^1^H-NMR spectra when the battery was left to rest at 0.2 V, corresponding to the full recovery of the diamagnetic MV^2+^ form and annihilation of in solution.

A single EPR spectrum during the charge or discharge periods of the battery exhibits a signal with multiplets, suggesting that the radical’s unpaired electron interacts with the neighboring nuclei via hyperfine coupling (Figure 3a). This interaction becomes even more apparent when considering the possible resonant structures, which illustrate that the unpaired electron in MV^+•^MV^+•^ is delocalized across the entire bipyridinium moiety (Figure 3b). To gain quantitative insight into the electronic structure of , we employed density functional theory (DFT) calculations using ORCA to predict its EPR parameters (Figure 3c). Consecutively, the computed parameters were used as the starting values for spectral simulations performed with EasySpin’s MATLAB toolbox (version 6.0.6).^31^ The model considered a total of 8 groups of nuclei. We chose 6 groups of protons and 2 non-equivalent nitrogens. This resulted in a total spin system described as (3H, 2H, 2H, 1N, 1N, 2H, 2H, 3H), where the number indicates the amount of magnetically equivalent nuclei. DFT-derived hyperfine couplings were used as the initial hyperfine coupling values for the simulation. To account for the fact that not all hyperfine splittings are fully resolved in the experimental spectra, we incorporated isotropic convolutional line broadening in the simulation, combining Gaussian and Lorentzian components; each was set to a value of 0.1 mT. Parameters such as the g-factor, hyperfine couplings, and line width were fitted to the experimental data by using the Nelder–Mead simplex method fitting routine. The model assumed isotropic conditions, which is suitable for liquid-phase.

**Figure 3:**
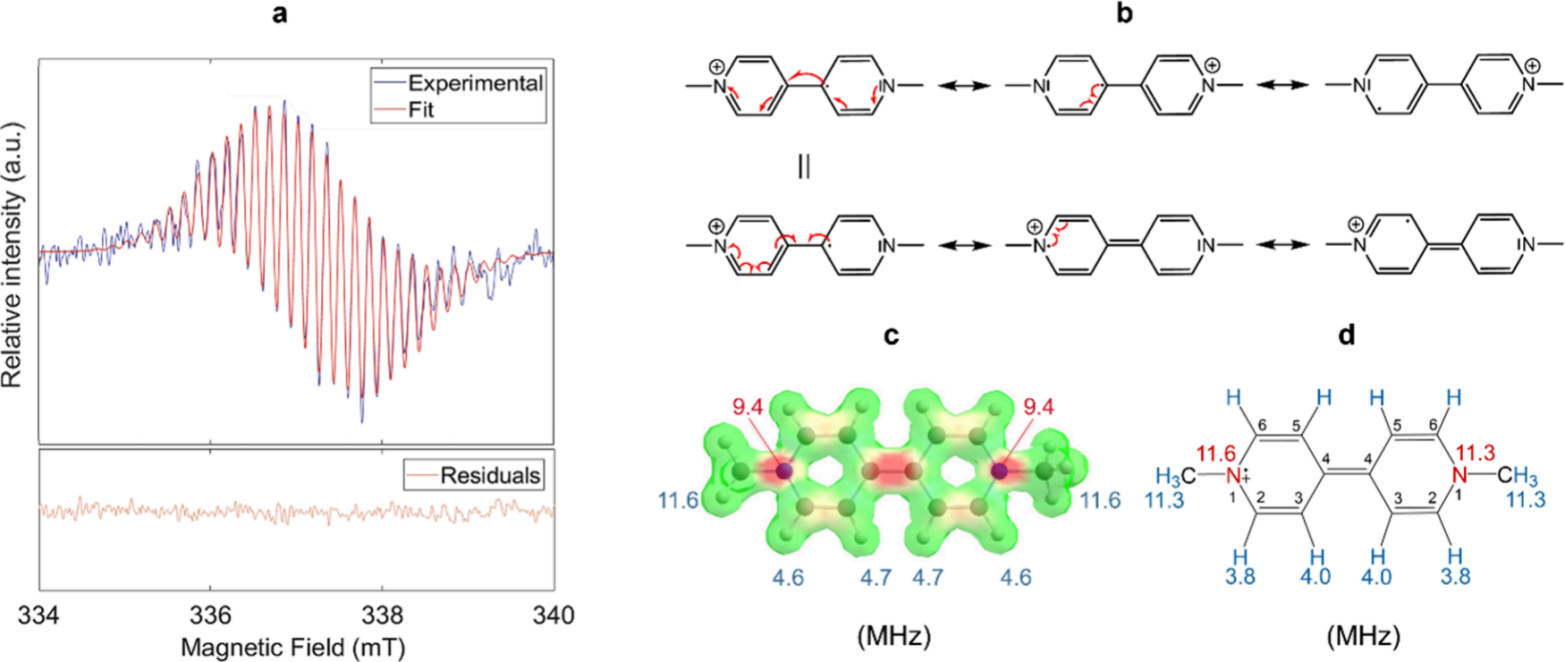
(a) Experimental EPR signal of MV^+•^ (blue trace), extracted from the first charge period at 22 min, and fitted simulation (red). The residuals of the fitting are shown below. Our spectra are centered at a *g*-factor of 2.00275. (b) Resonance structures of MV^+•^ (c) DFT-derived spin density surface map for MV^+•^, and the calculated isotropic hyperfine couplings (MHz, absolute values) for the active nuclei. (d) Hyperfine couplings in MHz obtained from the fitted EasySpin simulation of the experimental EPR spectrum. The main positions of the organic skeleton are labeled from 1 to 6.

The extracted hyperfine coupling constants (Figure 3d) show strong agreement with previously reported values for generated via photolysis or conventional electrochemical methods (see Table S3).^29,32–35^ Furthermore, the concentration of the radical cations during the experiment was determined following the spin-counting procedure (Figure S7).^6^ We did not use the Evans method to extract the radical concentration due to the chemical shift change being negligible, which is explained in more detail in the SI (see Figure S8).

The MV^+•^ radical cation gave a maximum concentration of approximately 3 mM for each charging step (Figure 3a). Based on the charge passed, i.e. Coulomb counting during the first half-cycle, and assuming full conversion, 8.5 mM of should have been generated from the 10 mM MV^2+^ starting material. However, spin-counting of the EPR signal reveals that only ∼35% of species are detected. This difference suggests that a significant portion of the reduced viologens is EPR-inactive.

We performed an additional experiment to determine whether oxygen quenching is the primary cause of this discrepancy following a reported protocol.^36^ This experiment consisted of a constant-current (CC) charging step, followed by a constant-voltage (CV) hold, and an open circuit potential (OCV) period. The evolution of the MV^+•^ concentration during this protocol was monitored in real time by EPR and is presented in Figure 4b. During the CC–CV charging, the radical concentration (blue curve) rose sharply (3.04 mM) as the voltage approached the cut-off limit, then plateaued at around 3 mM during the CV hold. Notably, no further increase in MV^+•^ concentration was observed despite continued voltage holding, which suggests that additional reduction of MV^2+^ did not increase the EPR-detectable radical pool. Furthermore, after switching to OCV conditions, the MV^+•^ concentration decreased gradually to 2.64 mM over 20 min. This loss of 0.40 mM of MV^+•^ corresponds to less than 6% of the 7 mM radical deficit relative to the expected 10 mM fully reduced state, indicating that oxygen quenching accounts for only a minor portion of the missing detectable radical concentration under the time scale of our experiment.

**Figure 4:**
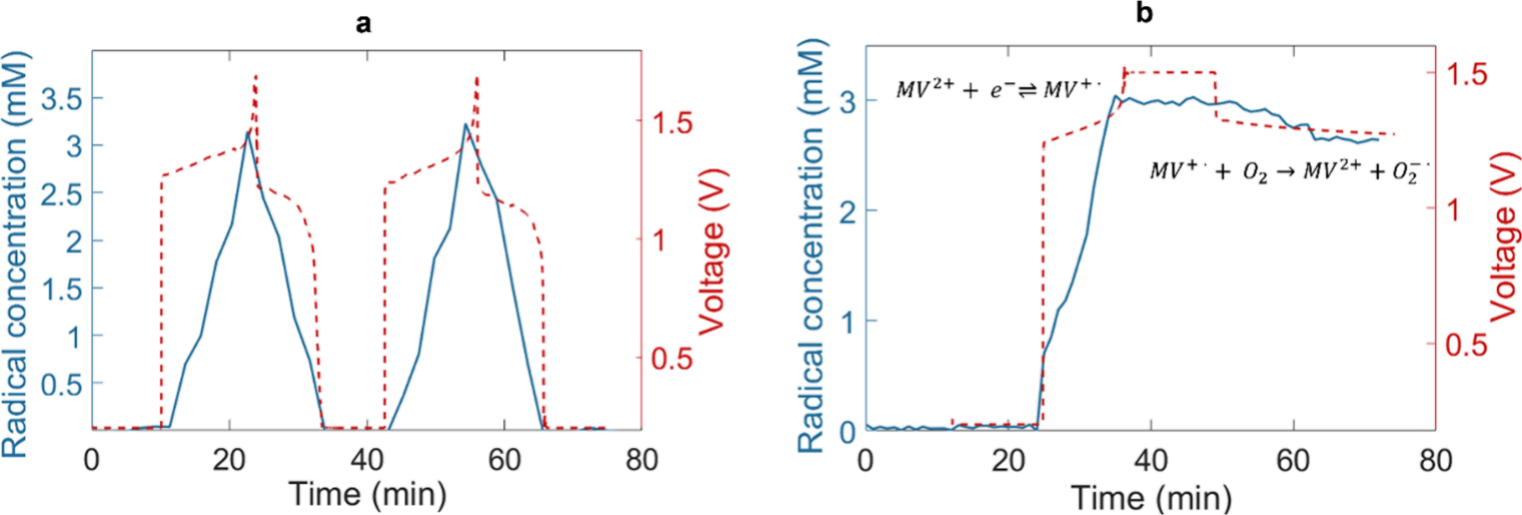
(a) Time evolution of MV^+•^ concentration (blue) and cell voltage (red) during the two charge and discharge cycles of the battery. (b) MV^+•^ concentration versus cell voltage during CC charging followed by a CV hold at 1.5 V, and OCV period. The rapid rise and plateau of the MV^+•^ concentration indicates a maximum EPR-detectable radical population during charging, consistent with dimer formation. The slow decay in MV^+•^ concentration during the OCV reflects minor quenching of MV^+•^ by residual oxygen. The annotations indicate the main redox reactions and the parasitic quenching pathway. In the latter, superoxide (O_2_^–•^) can react further to form hydrogen peroxide. Different cut-off voltages applied were due to the use of different membranes, FUMASEP membrane for (a) and PiperIon membrane for (b).

After ruling out a primary effect from oxygen, we attribute this difference to the occurrence of π-π stacking interactions between the formed MV^+•^ species, as has been proposed, resulting in dimerization and the spin-pairing of radicals, leading to the emergence of diamagnetism.^33^ As a result, the newly formed dimeric assemblies cannot be detected by EPR spectroscopy. Furthermore, such dimers will also be undetectable by NMR spectroscopy, as the radical fraction will be sufficient to obscure any proton resonances in the NMR spectrum due to considerable line broadening caused by intermolecular electron transfer. Nevertheless, the dimerization, likely coupled with intermolecular electron transfer among the dimers and monomers, warrants further investigation.

## Conclusions

In summary, we have demonstrated a novel benchtop *operando* NMR and EPR electrochemical methodology for characterizing the redox processes in a viologen-based RFB with sufficient sensitivity, spectral, and temporal resolution. The dual nature of the setup allowed us to track the ^1^H-NMR signals of MV^2+^ species during charge and discharge cycles while simultaneously detecting the formation and consumption of MV^+•^ radicals via EPR spectroscopy. The hyperfine coupling values are measured for the first time for the in-situ generated MV^+•^ in aqueous media, a system that has remained underexplored under battery operating conditions. Additionally, the low fraction of MV^+•^ generated during both charging cycles suggested the formation of dimers, which would result in reduced EPR signal intensity due to induced diamagnetism. This dimerization requires future investigation.

Our findings highlight the benchtop-based dual methodology as a robust, cost-effective, and space-efficient tool for characterizing organic RFBs. Beyond viologen chemistry, this approach holds broad applicability for investigating charge transfer mechanisms, structural changes, and radical formation in a variety of electrochemical systems. By establishing this coupled benchtop NMR-EPR as a powerful analytical tool for emerging RFB chemistries, we aim to advance the understanding and development of next-generation energy storage technologies.

## Supplementary Material





## Notes

Additional text, figures, and tables as described in the text (PDF)
eChem, ^1^H NMR, and EPR movie (MP4)

## ∥

Molecular Nanofabrication Group and Department of Molecules and Materials, MESA + Institute and Faculty of Science and Technology, University of Twente, 7500 AE Enschede, The Netherlands
