# Supplementary material for: Coupled Benchtop NMR and EPR Spectroscopy Reveals the Electronic Structure of Viologen Radicals in a Redox Flow Battery
Source: ACS Electrochem. 2025 Sep 23;1(10):1977–82. doi: 10.1021/acselectrochem.5c00194 (PMC12498384; doi:10.1021/acselectrochem.5c00194)
Supplement: Supplementary file 1 [file ec5c00194_si_001.pdf]

Supporting Information

Coupled Benchtop NMR and EPR Spectroscopy Reveals the Electronic Structure of Viologen Radicals in a Redox Flow Battery

Giu A. Silva Testa‡,[a] Mathijs A. Damhuis‡,[b] Tom Speelman,[c] Kim Baas,[a] Johannes A.A.W. Elemans*,[d] Evan Wenbo Zhao*[a]

[a] Magnetic Resonance Research Center, Institute for Molecules and Materials, Radboud University, 6525 AJ Nijmegen, The Netherlands. Corresponding E-mail: evanwenbo.zhao@ru.nl

[b] Magnetic Resonance Research Center, Institute for Molecules and Materials, Radboud University, 6525 AJ Nijmegen, The Netherlands. Present location: Molecular Nanofabrication Group and Department for Molecules and Materials, MESA + Institute and Faculty of Science and Technology, University of Twente, 7500 AE Enschede, The Netherlands

[c] Theoretical and Computational Chemistry, Institute for Molecules and Materials, Radboud University, 6525 AJ Nijmegen, The Netherlands.

[d] Spectroscopy and Catalysis, Institute for Molecules and Materials, Radboud University, 6525 AJ Nijmegen, The Netherlands. Corresponding E-mail: hans.elemans@ru.nl;

Contents

Materials 3

Synthesis of Methyl Viologen Dichloride 3

Cyclic voltammetry 7

Flow Battery Assembly 7

Flow System 7

Benchtop NMR capabilities 11

***Operando* NMR and EPR parameters** 12

EPR Calibration for Spin Counting 12

The Evans Method 13

DFT Calculations 14

Comparison with Reported Hyperfine Couplings in Different Solvents 15

References 16

Materials

4,4'-Bipyridine was obtained from TCI, and tetrabutylammonium chloride from Acros Organics, diethyl ether and ethanol were obtained from VWR chemicals, and all other chemicals were obtained from Sigma-Aldrich. For all reactions, reagents were used directly from the supplier. Reactions were followed by using thin-layer chromatography (TLC) on silica gel-coated plates (Merck 60 F254). NMR spectra were recorded at 298 K on a Bruker Avance III 400 spectrometer (400 MHz) equipped with a BBFO probe. ^{1}H-NMR chemical shifts (δ) are given in parts per million (ppm) and were referenced to tetramethylsilane (TMS, δH 0.00) or 3-(trimethylsilyl) propionic-2,2,3,3-d4 acid, sodium salt (δH 0.00) for D_2O. Data for ^{1}H-NMR spectra are reported as follows: chemical shift (multiplicity, coupling constant, integration). Multiplicities are abbreviated as s (singlet), d (doublet), t (triplet), q (quartet), m (multiplet), b (broad), and combinations thereof. NMR coupling constants are reported as J-values in Hertz (Hz).

Synthesis of Methyl Viologen Dichloride

Methyl viologen dichloride (MV^{2+}) was synthesized following a modified literature procedure.[1] 4,4'-Bipyridine (3.12 g, 20 mmol) was dissolved in 50 mL of acetonitrile and iodomethane (5 mL, 80 mmol) was added. The mixture was heated at 60 °C whilst stirring for 16 hours. After cooling, the resulting orange suspension was filtered and washed with several portions (6 x 25 mL) of diethyl ether to obtain crude methyl viologen diiodide (8.82 g, 20 mmol). The dichloride salt was subsequently obtained via the hexafluorophosphate salt. To an aqueous solution of methyl viologen diiodide (5.36 g, 12 mmol) a saturated solution of ammonium hexafluorophosphate was added until precipitation ceased, yielding an off-white solid with a slight yellow tinge. This crude intermediate was filtered and washed with several portions of water to remove the diiodide salt. Next, the hexafluorophosphate salt was dissolved in a small amount of acetonitrile to which a saturated solution of tetrabutylammonium chloride was added until precipitation ceased. This suspension was filtered, and the residue was washed with several portions of acetonitrile to remove the hexafluorophosphate salt. The resulting crude product was recrystallized from aqueous ethanol to yield pure MV^{2+} (2.45 g, 9.5 mmol, 79%). ^{1}H-NMR (400 MHz, DMSO-d6): δ 9.31 (d, 4H, ArH, ^3J = 7.1 Hz), 8.78 (d, 4H, ArH, ^3J = 7.1 Hz), 4.44 (s, 6H, CH_3) ppm. ^{13}C-NMR (101 MHz, DMSO-d6): δ 148.62 (ArC), 147.15 (ArC), 126.54 (ArC), 48.47 (CH_3) ppm. ^{19}F-NMR (101 MHz, DMSO-d6): NMR-silent, indicating the absence of PF_6^- ions.

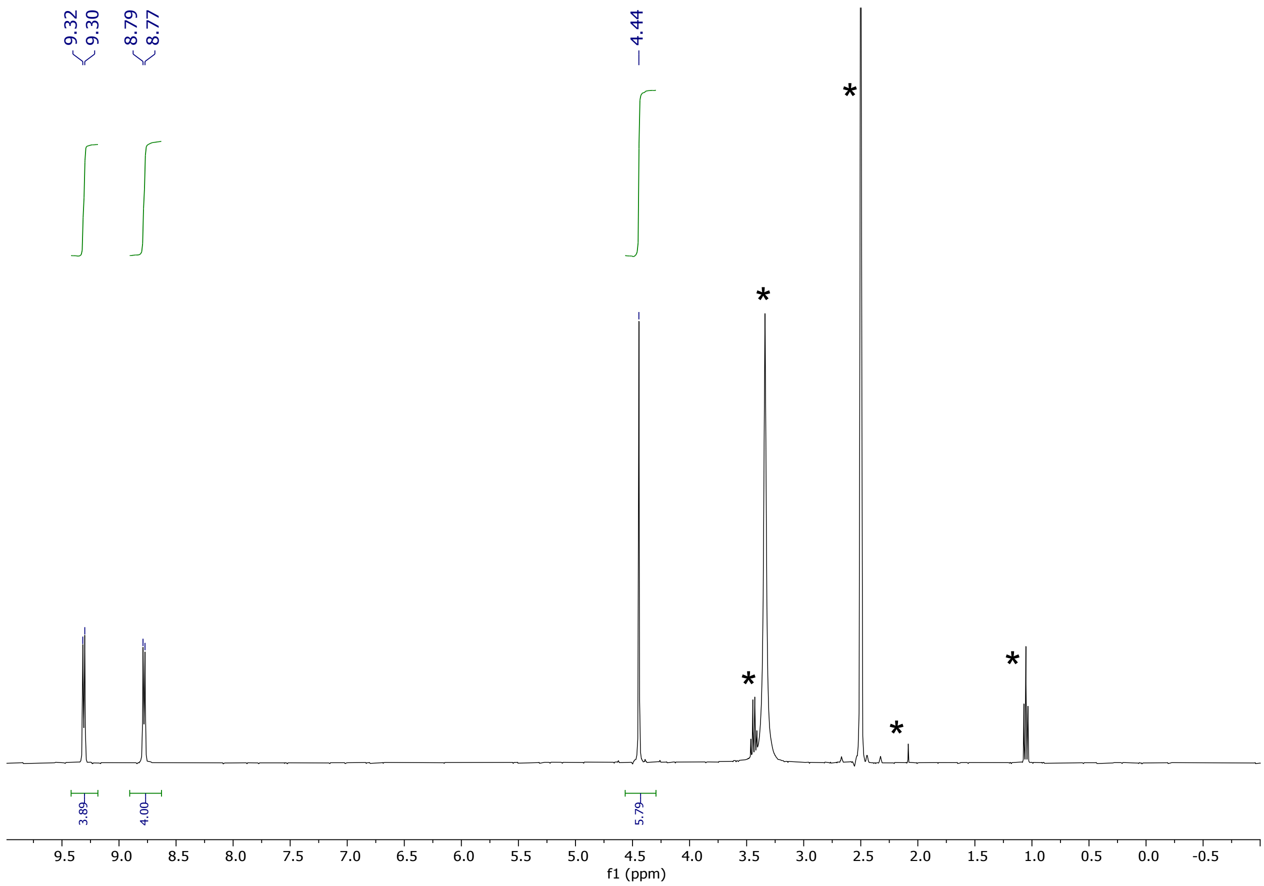

Supplementary Figure S1. ^1H-NMR spectrum (400 MHz, DMSO-d6) of MV^{2+}. The asterisks indicate residual solvent.

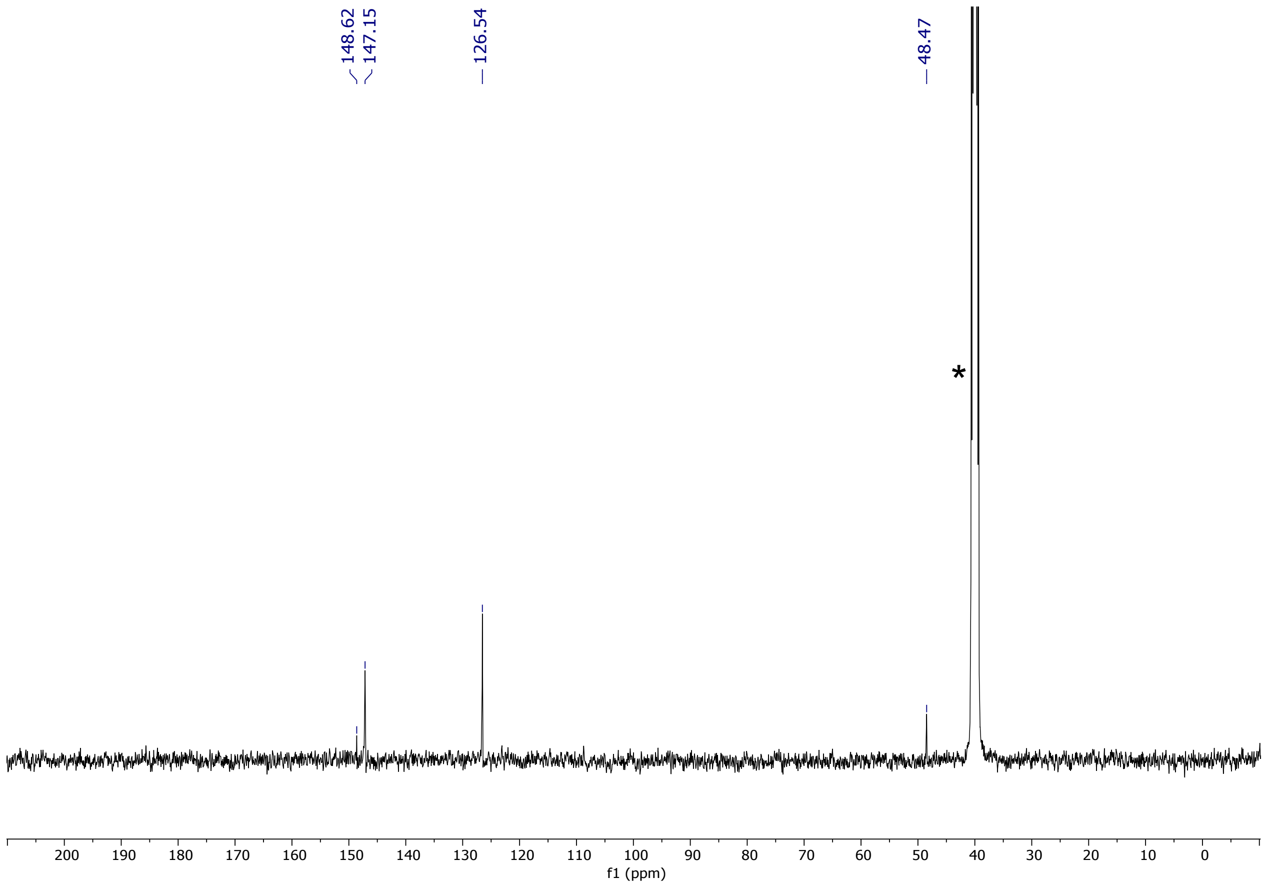

Supplementary Figure S2. ^{13}C-NMR spectrum (101 MHz, DMSO-d6) of MV^{2+}. The asterisk indicates residual solvent.

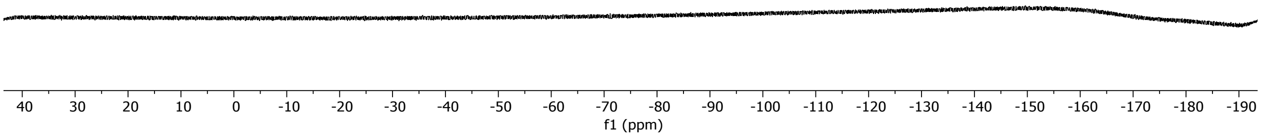

Supplementary Figure S3. ^{19}F-NMR spectrum (376 MHz, DMSO-d6) of MV^{2+}.

Cyclic voltammetry

The cyclic voltammograms of both electrolytes are shown in Fig. S4. For each CV, 4.0 mM of the redox-active compound was dissolved in 0.5 M of an aqueous sodium chloride solution; the scan rate was 50 mV/s for both measurements. A glassy carbon working electrode, a Pt counter electrode and an Ag/AgCl reference were used. The potential difference between MV^{2+} and TEMPOL results in a battery voltage of 1.31 V.

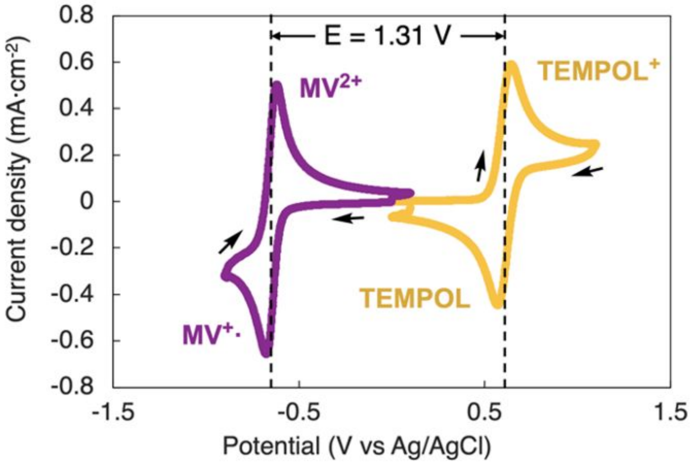

Supplementary Figure S4. Cyclic voltammograms of the one-electron redox conversions of MV^{2+} and TEMPOL under argon atmosphere. The arrows indicate the scanning direction.

Flow Battery Assembly

Experiments were conducted using a fuel cell from Fuel Cell Technologies Inc., which included two ultrahigh purity sealed graphite flow plates with serpentine flow patterns. The electrodes consisted of two layers of Sigracet 39 AA (SGL) carbon paper (5 cm^2 active area). FUMASEP (FAA-3-PK-130) was chosen as the anion exchange membrane, which was pre-treated by soaking it on a 0.1 M of aqueous KOH solution, a day prior to performing experiments. Only one experiment was performed with a different membrane (PiperION), which was activated by soaking in a 0.1M solution of NaCl before use. The carbon paper and the membrane were held up by Viton gaskets of 0.5 mm thickness. Gold-plated copper plates were used as the current collectors. A Gamry Frameworks Interface 1010E potentiostat was used to define the electrochemical sequence of the battery.

Flow System

Our *operando* NMR and EPR measurements would not be possible without the flow apparatus that allows the solutions to flow from the cell to the instrumentation. Selecting appropriate tubing and connectors can be challenging, here we provide a detailed list of the items needed to construct our flow system. The primary consideration is the selection of the pump, as it serves as the driving force behind the flow. We use Masterflex® L/S peristaltic pumps paired with Chem-Durance® Bio tubing. However, this tubing is incompatible with our fuel cell's inlets and outlets. By using the adequate connectors (Coned Fittings, see Table S1), the diameter of this tube can be reduced to accommodate 1/16'' flow tubing. This tubing should also seamlessly integrate with the connections to the NMR and EPR sampling tubes. Polyether ether ketone (PEEK) stands as an optimal material to use in these connections. PEEK presents exceptional mechanical and chemical resistance properties, even under extreme conditions, which are important considerations given the diverse chemistries encountered in the RFB community. Notably, IDEX Health & Science LLC. stands out as the only provider of a comprehensive range of PEEK connectors. An alternative material to PEEK is the use of stainless-steel connectors, such as Swagelok® fittings, which provide an exceptionally

leak-proof solution. However, they pose a slight challenge in terms of installation and removal due to their intended long-term placement, and they must be tightened with spanners. Hence, we recommend utilizing them for connections intended to be permanent rather than frequently removed. For this purpose, we only use these stainless-steel fittings for the in- and outlets- of the battery cell. The NMR sampling tube provided by the manufacturer (Bruker) was used without any modifications, as it was already compatible with 1/16'' perfluoro alkoxy (PFA) tubing. In contrast, the EPR sampling tube required adjustments to accommodate PEEK connectors. Specifically, modifications were made to the flat cell and the outer glass jacket (see Fig. S5). To enable the use of screw-on PEEK connectors, the outer ends of the sampling tube were carefully bored down, ensuring seamless integration of the connectors and the 1/16" tubing. The Supplementary Table 1 describes all the connectors mentioned here with the pertinent specifications to set up this flow system. A schematic illustration with the flow direction through the instrumentation is shown in Fig. S6.

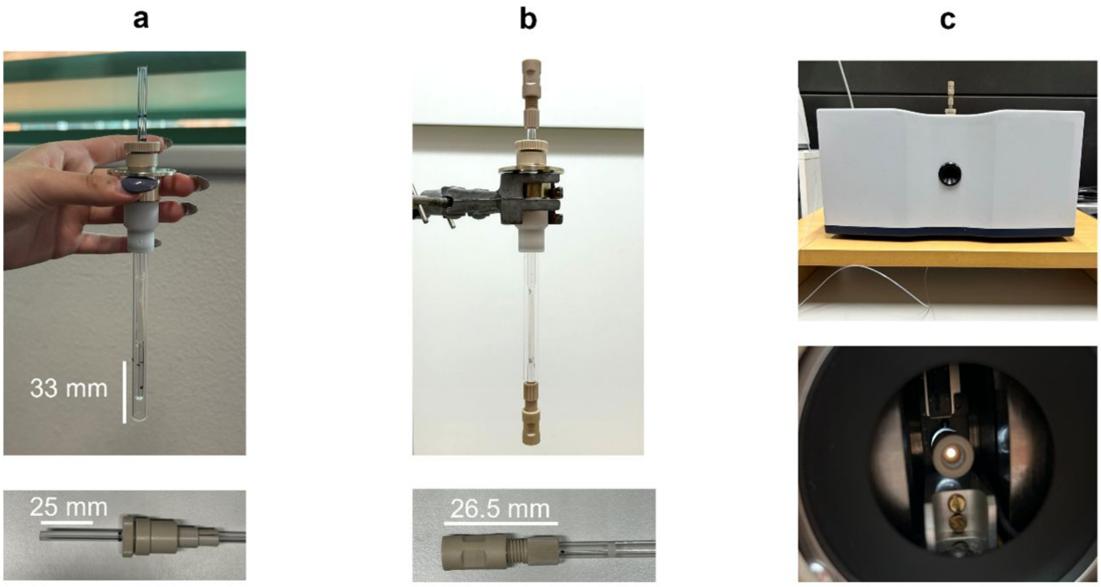

Supplementary Figure S5. (a) EPR sampling tube set-up prior to alterations. Both ends of the flat cell were bored down from a diameter of 5 mm to 4 mm. The bored down area had a length of 25 mm for each end of the flat cell. This ensured the fitting of P-330 nuts. Due to the bulkiness of the connectors, the outer glass tube that protects the flat cell had to be shortened by 33 mm. (b) EPR sampling tube set-up after the modifications, with both P-330 nuts and P-135 adapters inserted on each end of the flat cell. (c) Outside- and bottom-view of the modified EPR sampling tube once introduced into the instrument. The connectors protrude outside of the resonator, which allows for P-230 nuts to be screwed into. The latter allow for the insertion of 1/16'' tubing.

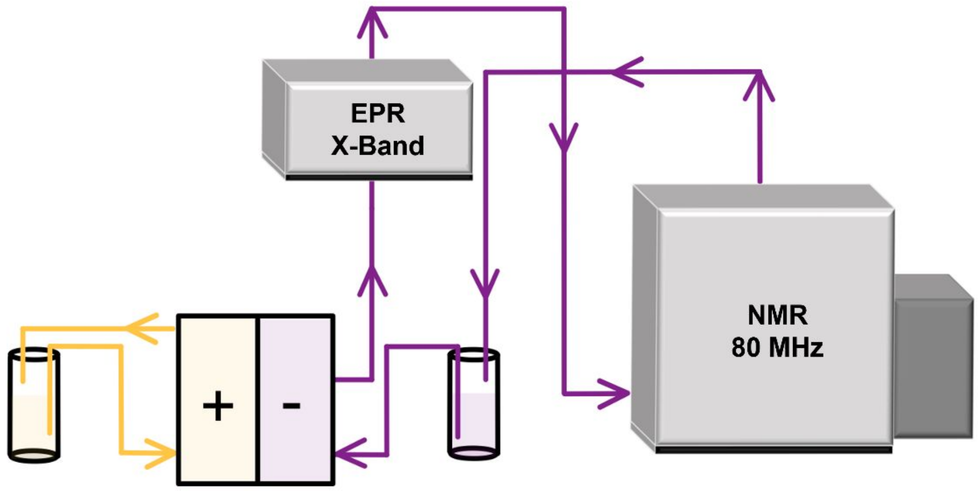

Supplementary Figure S6. Diagram representing the flow direction for both solutions, with the representative colours of TEMPOL (yellow) and $MV^{+\cdot}$ (purple). The total time of flight of the analyzed electrolyte is 1.1 min.

Supplementary Table S1. List of tubing and connectors used in our experimental flow system and their pertinent specifications.

Tubing	Specifications	Inner ⌀	Outer ⌀	Material	Description
Chem-Durance® Bio	644214-L4	1.6 mm	4.8 mm	-	Masterflex®. Compatible with Masterflex® Pump head L/S 14
PFA Tubing	1641L	1.55 mm	1/8"	PFA	IDEX Health & Science LLC. Compatible with inlet/outlet ports of some RFB cells.
PFA Tubing	1507L	1 mm	1/16"	PFA	IDEX Health & Science LLC. Compatible with Bruker Fourier80 sampling tube.

Connector	IDEX ID	Port config.	Tubing size	Material	Description
Coned Fitting	P-797	1.6 - 2.4 mm	1/16"	PEEK/EFTE	Connects rigid-walled tubing to soft-walled tubing. It inserts into the peristaltic pump tubing and connects to 1/16" tubing.
Flangeless Nut	P-330	-	1/8"	PEEK	Fits into 1/8" tubing and into our modified EPR sampling tube, which then can be connected to 1/16" tubing by using the P-135 adapter.
Flangeless Nut	P-230	-	1/16"	PEEK	Fits into 1/16" tube. Can be connected to P-135 or P-702.
Threaded Adapter	P-135	1/8" - 1/16”	-	PEEK	Connects P-330 nut with P-230 nut.
Union assembly	P-702	1/16" - 1/16”	-	PEEK	This assembly connects two P-230 nuts. Used for attaching separated 1/16" tubes, i.e., connecting the outlet tube of the RFB cell with the instrument’s sampling tube.

Connector	ID number	Port config.	Material	Description
Swagelok® Fittings	SS-200-6-1	1/16''-1/8''	Stainless steel	An assembly that connects tubing of two different sizes, can be used for the inlets and outlets of the RFB cell. Compatible with fuel cell hardware from Fuel Cell Technologies Inc.
Swagelok® Fittings	SS-100-6	1/16'' - 1/16''	Stainless steel	An assembly that connects tubing of the same size. Used for attaching separated 1/16" tubes, i.e., connecting the outlet tube of the RFB cell with the instrument’s sampling tube.

Benchtop NMR capabilities

Compared to traditional high-field (e.g., 400 MHz) NMR instruments, a benchtop system offers some key advantages. In the following Table S2, we list a comparison of different aspects and capabilities for both types of instruments.

Supplementary Table S2. Comparison of high-field (400 MHz) and benchtop (80 MHz) NMR systems for operando monitoring in RFB studies.

	**High-field (400 MHz)**	**Benchtop (80 MHz)**
**Resolution**	High spectral resolution (0.001 ppm), best for detailed structural analysis	Lower spectral resolution (~0.01 ppm), but sufficient for monitoring processes and quantifying small organic molecules
**Sensitivity**	Higher sensitivity, better SNR for dilute samples (sub-mM)	High sensitivity for typical electrolyte concentrations (~100 mM), well-resolved spectra achievable. Sufficient sensitivity for diluted samples (< 10 mM), longer scan times can improve detection limits when required
**Cost**	Purchase price \$500,000-\$1,000,000, high service contracts	Purchase price \$50,000-\$150,000, minimal running costs
**Space**	Requires dedicated space, vibration isolation, and shielding	Desktop-sized, can be installed in fume hoods or standard lab benches
**Compatibility**	Challenging to be coupled to other characterizations due to the cost and space constraints	Easy and straightforward to be coupled to other characterizations, particularly via flow
**Cryogenics**	Requires liquid helium and nitrogen for superconducting magnet, significant ongoing costs and logistics	Cryogen-free permanent magnet, no cryogen handling or replenishment required
**Operation**	Needs skilled NMR spectroscopist or technician, advanced training recommended	User-friendly interface, can be operated by non-specialists with basic training
**Maintenance**	Complex maintenance, requires service contracts, cryogen refills, specialized repairs	Simple upkeep, routine maintenance can be done in-house with minimal service visits

Operando NMR and EPR parameters

Inline ^{1}H-NMR spectra were acquired in D_2O using a benchtop Bruker Fourier80 spectrometer (80 MHz, 1.88 T). The spectral acquisition was achieved by conducting simple pulse-acquire experiments with a pulse angle of 90°. Prior to experiments, all electrolyte solutions underwent calibration to determine relevant NMR parameters and ensure sufficient resolution. Acquisition commenced upon complete filling of the flow cell with the electrolyte solution, which occurred after 50 s since the peristaltic pump initiates the flow through the system. Flow experiments require a residence time in the order of T_1 to allow the build-up of magnetization. Considering the NMR flow cell's volume of 0.396 mL, and a flow rate of 4 mL/min, the residence time of the sample in the detection region of the instrument was 6 s. The T_1 for each MV^{2+} proton and the residual solvent were determined: H_A = 1.4 s, H_B = 0.7 s, H_C= 0.7 s, HDO = 2.4 s. A recycle delay (d1) of 10 s, an acquisition time (aq) of 2 s, and 4 scans (ns) were chosen. The pulse sequence yielded a ^{1}H-NMR spectrum every 48 seconds ((d1 + aq) * ns = 48 s). Proton spectra were referenced to the chemical shift of residual H_2O at 4.74 ppm.

The EPR spectra were recorded on a Bruker Magnettech 5000 instrument (X-Band, 9.45 GHz) in continuous wave-mode at ambient temperature during battery operation. All EPR were acquired with the following parameters: modulation frequency = 100 kHz, magnetic field range = [330, 345] mT, modulation amplitude = 0.001 mT, accumulations = 1, sweep time = 60 s·scan^{-1}, and a microwave power = 0.5 mW.

EPR Calibration for Spin Counting

In order to extract the radical concentrations during the battery experiment, we first had to calibrate the system. The key principle is that the EPR signal intensity is directly proportional to the number of unpaired electrons (spins) present in the sample. Therefore, calibrating the instrument response with a well-characterized standard (such as TEMPOL) provides a reliable reference to convert signal intensity into radical concentration. This methodology is called *spin counting*. To ensure that results are comparable, the calibration and the $MV^{+\cdot}$ system should be acquired under the same EPR conditions and parameters.

Spin counting was performed to determine the radical concentration from EPR spectra by integrating the signal intensity (Fig. S7a). First, the first-derivative EPR spectra were acquired for TEMPOL solutions at known concentrations, ranging from 0.75 mM to 10 mM in D_2O. Each spectrum was numerically integrated to obtain the corresponding absorption spectrum (Fig. S7b), which was further integrated to yield the double integral. Since the double integral is directly proportional to the number of unpaired spins in the sample, we fitted the data by plotting the double integral as a function of concentration, showing a strong linear relationship (Fig. S7c). The radical concentration of $MV^{+\cdot}$ was determined by calculating the double integral and applying the calibration equation extracted earlier from TEMPOL samples.

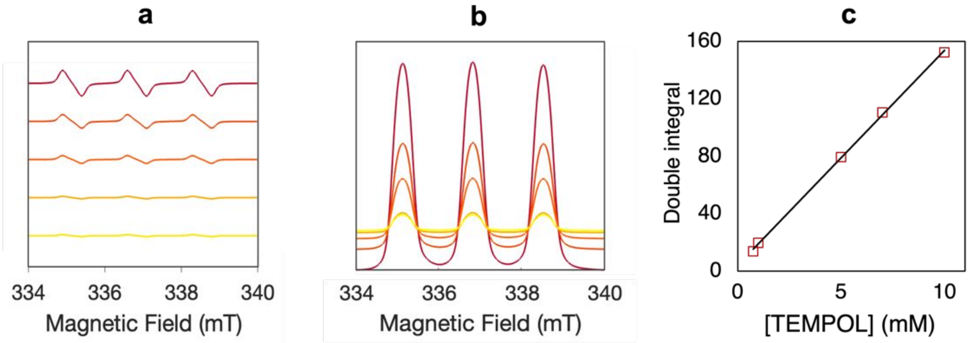

Supplementary Figure S7. (a) EPR spectra of TEMPOL solutions at different concentrations (0.75, 1 , 5, 7, 10 mM). The frequency of the microwave irradiation was 9.459275 GHz. (b) Absorption spectra obtained from the first integration of (a), the first derivative of the spectra. (c) Double integral of the EPR signal of five TEMPOL solutions at different concentrations (0.75, 1, 5, 7, 10 mM). The black line represents the result of a least-squares fit, with a slope of 14.96 mM^{-1} and R = 99.9%.

The Evans Method

The general form of the Evans equation is[2]:

$$\Delta\delta = \left(\frac{4\pi}{3} - \alpha\right)\frac{\Delta\chi_m}{V_m}$$

where $\Delta\delta$ is the chemical shift difference between an inert compound in the presence and absence of a paramagnetic ion, α is the shape-factor, $\Delta\chi_m$ is the molar magnetic susceptibility, and V_m is the molar volume of paramagnetic species. The equation can be expanded to explicitly relate the chemical shift to the concentration of the paramagnetic species:

$$\Delta\delta = \left(\frac{4\pi}{3} - \alpha\right)\frac{N_A\mu^2}{3K_BT}C_{para}$$

where N_A is Avogadro's number, μ is the effective magnetic moment of the paramagnetic species,k_B is Boltzmann's constant, T is the absolute temperature, and C_{para} is the molar concentration of the paramagnetic ion of interest. This relationship makes the Evans method a convenient tool for estimating the concentration of paramagnetic species, such as organic radicals, by measuring the chemical shift changes of a reference signal (e.g., the solvent HDO peak) during an ongoing process.

Our group has previously applied this approach successfully using a benchtop NMR setup.[3] However, in the present study, we did not employ the Evans method because the HDO chemical shift exhibited negligible changes throughout the experiment, indicating only a small concentration of radicals. To assess this, we estimated the chemical shift using the Evans method based on the radical concentration obtained by EPR spin counting (Fig. S8a), which is of the same order of magnitude as the observed variation in the full width at half maximum (FWHM) of the HDO peak (Fig. S8b). This result supports our experimental observation that the shift is too small to be distinguished reliably from baseline fluctuations and peak broadening. Therefore, EPR spin counting remained the most accurate and robust method for quantifying the radical concentration in our system. On the other hand, the accelerated T_2 relaxation, manifested as the line broadening, caused by the radicals can be used as an alternative method to estimate radical concentrations, which will be explored systematically in future studies.

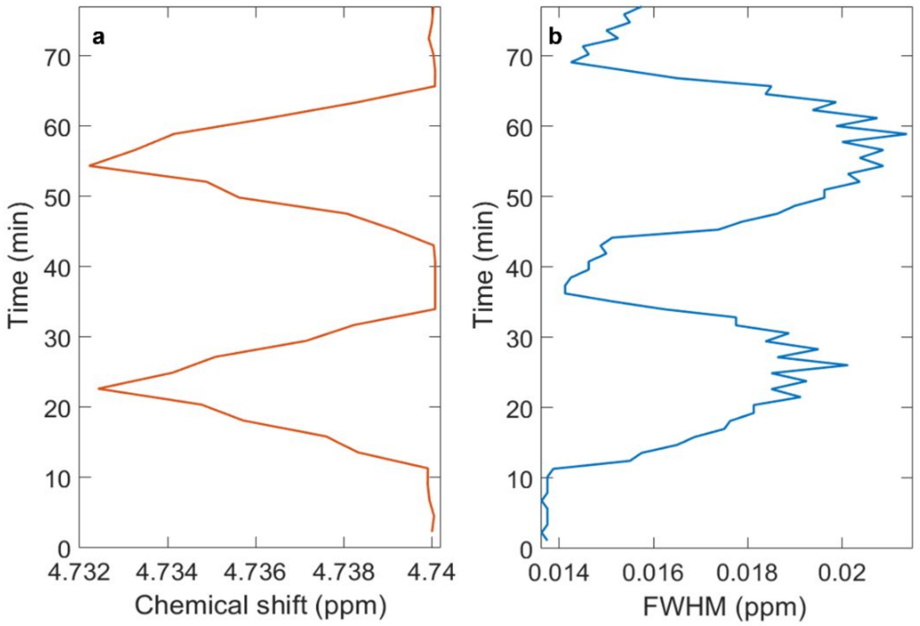

Supplementary Figure S8. (a) Estimated chemical shift of the HDO peak based on the Evans method using radical concentrations obtained from EPR spin counting. (b) Evolution of the HDO signal linewidth over time during battery cycling, shown as the FWHM, illustrating the observed peak broadening associated with radical formation. The chemical shift variation remains comparable to the FWHM of the HDO peak, justifying the use of EPR as the preferred quantification method.

DFT Calculations

The EPR parameters for $MV^{+\cdot}$ were also calculated through DFT calculations using the ORCA quantum chemistry package (version 5.0.3).[4] First, we performed a geometry optimization using the RI-B2PLYP functional,[5] with the def2-TZVP basis set,[6,7] plus the auxiliary def2-TZVP/C. The convergence threshold was set using "TIGHTOPT". The optimised geometry is listed below:

28
optimized viologen

N	3.54055158056164	0.00028034107210	0.02913349061328
C	2.84487475899956	1.17365010184522	0.01844678546729
C	1.48286061568975	1.20056784154713	0.00894337079561
C	2.84498156146688	-1.17325556456540	0.01903020918847
C	1.48302026315998	-1.20035948832657	0.00947825142963
C	0.71291417338494	0.00006900784336	0.00376460850951
C	-0.71291567989056	-0.00007414543077	-0.00379507814247
C	-1.48302422800091	1.20035418438119	-0.00922061747909
C	-2.84498487185731	1.17324900206145	-0.01876469314153
C	-1.48285994886308	-1.20057270002059	-0.00926208504175
C	-2.84487398169513	-1.17365525935615	-0.01876874493156
N	-3.54055330151797	-0.00028548174572	-0.02916379696121
C	5.00367933588986	-0.00033190421093	-0.03392015342112
C	-5.00368232317602	0.00031491773503	0.03386678115618
H	-5.33916781130612	0.01079266870311	1.07056661133080
H	-5.38367292906554	-0.88903754596985	-0.46105331546373
H	-5.38407542165843	0.87921865120444	-0.47924566083164
H	5.33914812252645	-0.01096832633767	-1.07062363598957
H	5.38407650864794	-0.87915985473873	0.47931860174949
H	5.38368136141232	0.88909337410941	0.46086136070204
H	-1.01418862255513	-2.17176621228047	-0.00980917185044
H	-3.43772190903341	-2.07602277409173	-0.02211435197933
H	-1.01444463582975	2.17159466712578	-0.00964687158333

H -3.43805371959886 2.07549303952595 -0.02208254275689
H 1.01443679722795 -2.17159777537265 0.01021612028905
H 3.43804758837123 -2.07550047082413 0.02260279113444
H 3.43772504602371 2.07601712872289 0.02153758212509
H 1.01419167068600 2.17176257739432 0.00918415508277

The g-tensor and hyperfine couplings were then calculated using the optimised geometry. For all nuclei the isotropic, dipolar and 2nd order spin-orbit coupling contributions to the hyperfine coupling as well as the electric field gradient and electron density at the nucleus were requested. Again, the RI-B2PLYP functional was used but now with the EPR-III basis set,[8,9] combined with the "AutoAUX" flag for automatic generation of auxiliary basis functions.[10] Relativistic effects were included via the RI-SOMF(1X) method.[11,12] Additionally, the "SlowConv" flag was set.

Comparison with Reported Hyperfine Couplings in Different Solvents

Parameters obtained through DFT calculations were used as starting parameters for spectral simulations using EasySpin.[13] Table S3 compares our extracted hyperfine coupling constants, from the EasySpin fitting procedure, with those from previous reports on $MV^{+\bullet}$, demonstrating similar spin density distribution across different solvents, showing negligible solvent effect.

Supplementary Table S3. Reported hyperfine couplings (A) for each type of nuclei of $MV^{+\bullet}$ in this current work and the literature. The generation method for the radicals in each experiment has been specified as PL (Photolysis) or EC (Electrochemical). Nuclei were labeled corresponding to their position within the bipyridinium moiety.(*) Values were not assigned to each type of protons in this publication.

Method	**RFB (This study)**	EC	PL	PL	PL
Solvent	Water	Water	Ethanol	Methanol	Methanol
Hyperfine couplings	A (MHz)	A (MHz)[14] (*)	A (MHz)[15]	A (MHz)[16]	A (MHz)[17]
(H) 2,6	3.8	3.7	3.7	3.7	3.7
(H) 3,5	4.0	4.4	4.4	4.4	4.3
(H) $-CH_3$	11.3	11.2	11.1	11.2	11.1
N	11.6	11.8	11.8	11.8	11.8

References

(1) Alotto, P.; Guarnieri, M.; Moro, F. Redox Flow Batteries for the Storage of Renewable Energy: A Review. *Renewable and Sustainable Energy Rev.* **2014**, *29*, 325–335. https://doi.org/10.1016/j.rser.2013.08.001.

(2) Evans, D. F. The Determination of the Paramagnetic Susceptibility of Substances in Solution by Nuclear Magnetic Resonance. *J. Chem. Soc.* **1959**, 2003–2005.

(3) Wu, B.; Aspers, R. L. E. G.; Kentgens, A. P. M.; Zhao, E. W. Operando Benchtop NMR Reveals Reaction Intermediates and Crossover in Redox Flow Batteries. *J. Magn. Res.* **2023**, *351*, 107448. https://doi.org/10.1016/j.jmr.2023.107448.

(4) Neese, F. Software Update: The ORCA Program System—Version 5.0. *WIREs Comput. Mol. Sci.* **2022**, *12* (5), e1606. https://doi.org/10.1002/wcms.1606.

(5) Grimme, S. Semiempirical Hybrid Density Functional with Perturbative Second-Order Correlation. *The Journal of Chemical Physics* **2006**, *124* (3), 034108. https://doi.org/10.1063/1.2148954.

(6) Weigend, F.; Ahlrichs, R. Balanced Basis Sets of Split Valence, Triple Zeta Valence and Quadruple Zeta Valence Quality for H to Rn: Design and Assessment of Accuracy. *Phys. Chem. Chem. Phys.* **2005**, *7* (18), 3297. https://doi.org/10.1039/b508541a.

(7) Weigend, F. Accurate Coulomb-Fitting Basis Sets for H to Rn. *Phys. Chem. Chem. Phys.* **2006**, *8* (9), 1057. https://doi.org/10.1039/b515623h.

(8) BARONE, V. Structure, Magnetic Properties and Reactivities of Open-Shell Species From Density Functional and Self-Consistent Hybrid Methods. In *Recent Advances in Density Functional Methods*; Recent Advances in Computational Chemistry; WORLD SCIENTIFIC, 1995; Vol. Volume 1, pp 287–334. https://doi.org/10.1142/9789812830586_0008.

(9) Rega, N.; Cossi, M.; Barone, V. Development and Validation of Reliable Quantum Mechanical Approaches for the Study of Free Radicals in Solution. *. J. .Chem.l Phys.***1996**, *105* (24), 11060–11067. https://doi.org/10.1063/1.472906.

(10) Stoychev, G. L.; Auer, A. A.; Neese, F. Automatic Generation of Auxiliary Basis Sets. *J. Chem. Theory Comput.* **2017**, *13* (2), 554–562. https://doi.org/10.1021/acs.jctc.6b01041.

(11) Heß, B. A.; Marian, C. M.; Wahlgren, U.; Gropen, O. A Mean-Field Spin-Orbit Method Applicable to Correlated Wavefunctions. *Chem. Phys. Lett.* **1996**, *251* (5–6), 365–371. https://doi.org/10.1016/0009-2614(96)00119-4.

(12) Neese, F. Efficient and Accurate Approximations to the Molecular Spin-Orbit Coupling Operator and Their Use in Molecular g-Tensor Calculations. *.J. Chem. Phys.***2005**, *122* (3), 034107. https://doi.org/10.1063/1.1829047.

(13) Stoll, S.; Schweiger, A. EasySpin, a Comprehensive Software Package for Spectral Simulation and Analysis in EPR. *J. Magn. Res.* **2006**, *178* (1), 42–55. https://doi.org/10.1016/j.jmr.2005.08.013.

(14) Neukermans, S.; Samanipour, M.; Vincent Ching, H. Y.; Hereijgers, J.; Van Doorslaer, S.; Hubin, A.; Breugelmans, T. A Versatile *In-Situ* Electron Paramagnetic Resonance Spectro-electrochemical Approach for Electrocatalyst Research. *ChemElectroChem* **2020**, *7* (22), 4578–4586. https://doi.org/10.1002/celc.202001193.

(15) Johnson, C. S.; Gutowsky, H. S. High-Resolution ESR Spectra of Photochemically Generated Free Radicals: The Viologens. *J. .Chem. Phys* **1963**, *39* (1), 58–62. https://doi.org/10.1063/1.1734033.

(16) Evans, A. G.; Evans, J. C.; Baker, M. W. Study of Bipyridyl Radical Cations. Part 5. Effect of Structure on the Dimerisation Equilibrium. *J. Chem. Soc., Perkin Trans. 2* **1977**, No. 13, 1787. https://doi.org/10.1039/p29770001787.

(17) Rieger, A. L.; Rieger, P. H. Magnetic Resonance Studies of Some Bipyridylium Dications and Cation Radicals. *J. Phys. Chem.* **1984**, *88* (24), 5845–5851. https://doi.org/10.1021/j150668a021.
